# Paclitaxel with or without trametinib or pazopanib in advanced wild-type BRAF melanoma (PACMEL): a multicentre, open-label, randomised, controlled phase II trial

**DOI:** 10.1093/annonc/mdy500

**Published:** 2018-11-14

**Authors:** V Urbonas, D Schadendorf, L Zimmer, S Danson, E Marshall, P Corrie, M Wheater, E Plummer, C Mauch, C Scudder, M Goff, S B Love, S B Mohammed, M R Middleton

**Affiliations:** 1 Early Phase Clinical Trials Unit, Oxford University Hospitals NHS Foundation Trust, Oxford, UK; 2 National Cancer Institute, Vilnius, Lithuania; 3 Department of Dermatology, University Hospital Essen, West German Cancer Centre, University Duisburg-Essen, Essen, Germany; 4 The German Cancer Consortium, Essen, Germany; 5 Department of Oncology, Sheffield Experimental Cancer Medicine Centre, Weston Park Hospital, Sheffield, UK; 6 Department of Oncology, Clatterbridge Cancer Centre, Wirral, UK; 7 Department of Oncology, Addenbrookes Hospital, Cambridge, UK; 8 Department of Oncology, Southampton General Hospital, Southampton, UK; 9 Department of Oncology, Freeman Hospital, Newcastle upon Tyne, UK; 10 Köln Universitätsklinik, Köln, Germany; 11 Oncology Clinical Trials Office, University of Oxford, Oxford, UK; 12 Centre for Statistics in Medicine, University of Oxford, Oxford, UK; 13 Department of Oncology, NIHR Oxford Biomedical Research Centre, University of Oxford, Oxford, UK

**Keywords:** phase II, melanoma, BRAF wild-type, pazopanib, trametinib

## Abstract

**Background:**

Advanced melanoma treatments often rely on immunotherapy or targeting mutations, with few treatment options for wild-type *BRAF* (*BRAF*-wt) melanoma. However, the mitogen-activated protein kinase pathway is activated in most melanoma, including *BRAF*-wt. We assessed whether inhibiting this pathway by adding kinase inhibitors trametinib or pazopanib to paclitaxel chemotherapy improved outcomes in patients with advanced *BRAF*-wt melanoma in a phase II, randomised and open-label trial.

**Patients and methods:**

Patients were randomised (1 : 1 : 1) to paclitaxel alone or with trametinib or pazopanib. Paclitaxel was given for a maximum of six cycles, while 2 mg trametinib and 800 mg pazopanib were administered orally once daily until disease progression or unacceptable toxicity. Participants and investigators were unblinded. The primary end point was progression-free survival (PFS). Key secondary end points included overall survival (OS) and objective response rate (ORR).

**Results:**

Participants were randomised to paclitaxel alone (*n *=* *38), paclitaxel and trametinib (*n *=* *36), or paclitaxel and pazopanib (*n *=* *37). Adding trametinib significantly improved 6-month PFS [time ratio (TR), 1.47; 90% confidence interval (CI) 1.08–2.01, *P *=* *0.04] and ORR (42% versus 13%; *P *=* *0.01) but had no effect on OS (*P *=* *0.25). Adding pazopanib did not benefit 6-month PFS; (TR 1.36; 90% CI 0.96–1.93; *P *=* *0.14), ORR, or OS. Toxicity increased in both combination arms.

**Conclusion:**

In this phase II trial, adding trametinib to paclitaxel chemotherapy for *BRAF*-wt melanoma improved PFS and substantially increased ORR but did not impact OS.

This study was registered with the EU Clinical Trials Register, EudraCT number 2011-002545-35, and with the ISRCTN registry, number 43327231.

**Key Message**

Our study indicates that addition of trametinib to paclitaxel chemotherapy was associated with an improvement in progression-free survival and increase in overall response rate in patients with *BRAF* wild-type melanoma. As in the NEMO study, comparing binimetinib with dacarbazine in *NRAS* mutant melanoma, this did not translate into an overall survival benefit. It appears that MEK inhibitors have some activity in *BRAF*-wt melanoma, but that this is not sustained.

## Introduction

Although substantial progress has been achieved in the management of unresectable and metastatic melanoma, it remains a fatal disease. Impressive anticancer activity has been shown by kinase inhibitors, which target aberrant signalling in the 50% of melanomas with *BRAF* mutations, and antibodies that bind to the immune checkpoints cytotoxic T-lymphocyte antigen 4 (CTLA-4) or programmed death 1 (PD1) [1–3]. However, melanoma patients with wild-type *BRAF* (*BRAF*-wt) have fewer treatment choices, as they do not benefit from BRAF inhibitor therapy. 

Extracellular-signal-regulated kinase (ERK) is constitutively active in all melanoma, irrespective of its mutation status [4]. ERK is part of the mitogen-activated protein kinase (MAPK) pathway, which is activated by an upstream kinase called MAPK kinase, or MEK. Activating ERK through the MAPK pathway promotes melanoma cell growth and resistance to taxane chemotherapy, the latter by inhibiting apoptosis [5]. In pre-clinical *BRAF*-wt melanoma models, co-administering a taxane and a MEK inhibitor substantially induces tumour regression and apoptosis [6, 7]. A clinical trial of the taxane docetaxel with the MEK inhibitor selumetinib reported a higher response rate than with chemotherapy alone [8].

Pazopanib is an orally bioavailable, multi-target tyrosine kinase inhibitor of vascular endothelial growth factor receptors 1, 2, and 3 (VEGFR1, 2, 3), platelet-derived growth receptors α and β (PDGFR α and β), and stem cell factor receptor (c-KIT). As well as its antiangiogenic effects, pazopanib inhibits MEK and ERK activation at clinically relevant doses [9]. Trametinib is a reversible, highly selective, allosteric inhibitor of MEK1/2 activation. Both pazopanib and trametinib can safely be given with weekly paclitaxel chemotherapy at the full monotherapy dose and showed promising activity in *BRAF*-wt melanoma in early clinical studies [10].

We therefore undertook the PACMEL phase II trial to better assess the benefits and safety of adding trametinib or pazopanib to paclitaxel chemotherapy in *BRAF*-wt unresectable or metastatic melanoma. 

## Methods

### Participants

This randomised, multicentre phase II trial was conducted in 26 centres in the UK and Germany. Participants were 18 years or older with measurable unresectable *BRAF*-wt stage 3 or 4 melanoma, an Eastern Cooperative Oncology Group (ECOG) score of 0 or 1, and acceptable haematological, renal, and hepatic function. Patients were excluded if they had received a prior MEK inhibitor or taxane, had grade ≥2 peripheral neuropathy, had undergone recent systemic therapy or radiotherapy, or had a recent history of another active malignancy. Patients with mucosal or ocular melanoma or with ocular disease that predisposed them to central serous retinopathy or retinal vein occlusion were excluded.

The study protocol was approved by the independent ethics committee or independent review board at each study centre. All patients provided written consent to participate before screening procedures. The study was conducted in accordance with the Declaration of Helsinki and Good Clinical Practice guidelines.

### Study design and treatment

PACMEL phase II was an open-label, multicentre, and randomised trial that evaluated paclitaxel in combination with trametinib or pazopanib. Eligible patients were randomised 1 : 1 : 1 to receive single-agent paclitaxel or paclitaxel combined with trametinib or pazopanib, stratifying for *NRAS* mutation status, lactate dehydrogenase level (LDH; elevated versus within normal limits), and prior therapy for metastatic melanoma (yes versus no), using minimisation with a random element of 0.8. Randomisation was administered by a central trials unit to maintain allocation concealment. Participants, investigators, and outcome assessors were unblinded to treatment allocation.

Paclitaxel (80 mg/m^2^ in the single-agent paclitaxel and paclitaxel–trametinib arms, 65 mg/m^2^ in the paclitaxel–pazopanib arm) was administered intravenously on days 1, 8, and 15 of each cycle for up to a maximum of six cycles. Trametinib (2 mg, determined during PACMEL phase I) or pazopanib (800 mg) were administered orally once daily until disease progression or unacceptable toxicity. Dose reductions of paclitaxel, trametinib, and pazopanib were permitted for toxicity.

### End points

The primary end point was progression-free survival (PFS) using Response Evaluation Criteria in Solid Tumours (RECIST) v1.1. Secondary end points included overall survival (OS), objective response rate (ORR), 6-month progression-free percentage, safety, and tolerability. Adverse events (AEs) were graded according to the Common Toxicity Criteria for Adverse Events (CTCAE) version 4.03.

### Assessments

At screening, prospective participants gave a medical history and underwent a full physical examination; ophthalmological evaluation; pregnancy test; 12-lead ECG; echocardiography; computed tomography (CT) of the head, chest, abdomen, and pelvis; urinalysis; haematology; coagulation and blood chemistry testing; and *NRAS* and *BRAF* mutation assessment.

Participants underwent a targeted physical assessment, AE documentation, urinalysis, and full blood chemistry and haematology testing before each paclitaxel dosing. Participants on trametinib or pazopanib underwent echocardiography at baseline and weeks 4 and 12, then every 12 weeks whilst on treatment. All participants underwent repeat CT assessments at weeks 7, 15, and 23, then every 3 months until disease progression. All participants underwent additional ophthalmological examination as clinically indicated.

### Statistical analysis

We planned to recruit 120 patients, randomised 1 : 1 : 1 to allow independent comparison of each combination arm with single-agent paclitaxel. PFS analysis was planned after 58 events in each comparison, giving 80% power to detect a hazard ratio of 0.57, with a one-sided *α* level of 0.10. Recruitment was significantly slower than anticipated, allowing a revised minimum of 104 patients to deliver the required number of events. All statistical analyses were pre-specified in the statistical analysis plan that was signed off before the data analysis.

All efficacy analyses were carried out on an intention-to-treat basis, analysing all randomised patients according to the treatment arm to which they were randomised. Median follow-up time was calculated using the reverse Kaplan–Meier method. Patients who withdrew consent for further follow-up were censored at the time of withdrawal.

PFS was defined as the time from the date of randomisation to the date of progression or death from any cause, whichever came first. PFS at 6 months was defined as the Kaplan–Meier estimate percentage of participants who were progression-free at 6 months, with a 90% confidence interval (CI). Participants without an event were censored at the time of their last assessment. OS was defined as the time from the date of randomisation to the date of death. Participants without an event were again censored at the date of their last visit. ORR was defined as the best overall response for each participant, portrayed as the proportion of participants achieving a complete or partial response out of all randomised participants.

The PFS and OS were compared between treatment arms using Cox regression analysis, adjusting for the stratification variables. The proportional hazard assumption was then checked using Schoenfeld residuals. If the assumption was not met, then the Cox regression results were not considered valid and were not to be reported. Instead, the analysis used an accelerated failure time (AFT) model as specified in the Statistical Analysis Plan [11] (supplementary Figure S1, available at *Annals of Oncology* online) AFT results are reported as a time ratio (TR) with 90% CI.

The ORR was compared between treatment groups using the *χ*^2^ test and odds ratios. Safety analyses were carried out on patients who received at least one treatment dose. AEs grade ≥3 and serious AEs (SAEs) between treatment groups were compared using the *χ*^2^ test.

A sensitivity analysis was carried out on the per-protocol population to examine the robustness of the intention-to-treat analysis conclusions. A sub-group analysis was planned comparing outcomes in the *NRAS* mutant and wild-type subgroups.

STATA v14.1 (StataCorp, College Station, TX) was used in all analyses. All reported *P*-values are two-sided for the primary and secondary analyses.

## Results

### Participants and treatment

**Figure 1.**
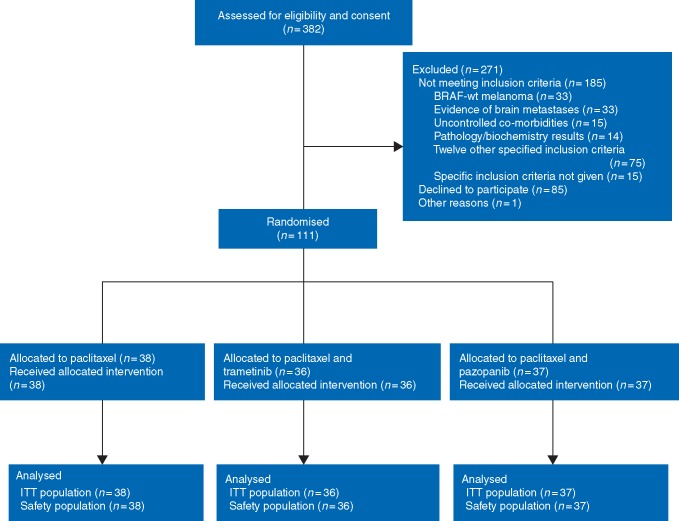
CONSORT diagram.

Between April 2012 and March 2016, 382 patients were considered for the trial at 26 sites in the UK and Germany. We randomised 111 patients to treatment (paclitaxel, *n *=* *38, paclitaxel–trametinib, *n *=* *36, and paclitaxel–pazopanib, *n *=* *37). Although smaller than the calculated sample size, this sample provided the required number of events to achieve the power calculated and all 111 were included in the efficacy and safety analyses (Figure 1). The most common reasons for non-participation were patients declining to participate (85 patients), melanoma with *BRAF* mutation (33 patients), evidence of brain metastases (33 patients), and uncontrolled co-morbidities (15 patients). Participant demographics and baseline clinical characteristics (Table 1) were well balanced across the three arms. Most (64%) of the patients were treatment-naïve.

**Table 1.** Patient characteristics at baseline

Characteristics	Paclitaxel (*n *=* *38)	Paclitaxel+trametinib (*n *=* *36)	Paclitaxel+pazopanib (*n *=* *37)
Age, years, median (range)	64 (35–80)	60 (27–80)	66 (41–80)
Gender male, *n* (%)	27 (71)	23 (64)	25 (68)
Ethnicity, *n* (%)			
White	35 (92)	33 (92)	34 (92)
Other ethnic groups	2 (5)	2 (6)	2 (5)
Not given	1 (3)	1 (3)	1 (3)
ECOG performance score, *n* (%)			
0	21 (55)	23 (64)	25 (68)
1	17 (45)	13 (36)	12 (32)
Disease stage at entry, *n* (%)			
IV	38 (100)	34 (94)	34 (92)
Unresectable stage III	0 (0)	2 (6)	3 (8)
Stratification variables, *n* (%)			
Prior therapy	13 (34)	12 (33)	14 (38)
LDH within normal range	19 (50)	18 (50)	18 (49)
NRAS mutant	14 (37)	14 (39)	18 (49)

*n*, number of patients in a group; LDH, lactate dehydrogenase; ECOG, Eastern Cooperative Oncology Group.

Paclitaxel was given for a median 3, 6, and 4 cycles as a single agent, with trametinib, and with pazopanib, respectively. Six cycles of paclitaxel treatment were completed by 19% of participants as a single agent, 42% in the paclitaxel–trametinib arm, and 20% in the paclitaxel–pazopanib arm. The main reasons for discontinuing paclitaxel treatment were progressive disease (62% single-agent paclitaxel, 49% paclitaxel–trametinib, and 43% paclitaxel–pazopanib) and toxicity (8% single-agent paclitaxel, 6% paclitaxel–trametinib, and 32% paclitaxel–pazopanib). The inhibitor agent was taken for more than 4 months by 17 (47%) participants on trametinib and 11 (30%) participants on pazopanib.

### Efficacy

After 95 events, the median PFS was 3.4 months (90% CI 2.0–3.8) with paclitaxel alone, 5.2 months (90% CI 3.7–7.0) with paclitaxel and trametinib, and 5.3 months (90% CI 3.4–6.4) with paclitaxel and pazopanib (Table 2). There were more than 58 events in each of the pairwise comparisons, as required for the calculated power.

**Table 2.** Summary of clinical outcomes by treatment arm

End point	Paclitaxel (*n *=* *38)	Paclitaxel+trametinib (*n *=* *36)	Paclitaxel+pazopanib (*n *=* *37)
Progression-free survival (months)			
Median duration (90% CI)	3.4 (2.0–3.8)	5.2 (3.7–7.0)	5.3 (3.4–6.4)
TR^a^ (90% CI)	1.0	1.47 (1.08–2.01) *P* = 0.04	1.36 (0.96–1.93) *P* = 0.14
Progression-free survival rate at 6 months			
Percentage (90% CI)	27 (16–40)	39 (26–52) *P* = 0.27	41 (28–55) *P* = 0.18
Overall survival (months)			
Median duration (90% CI)	10.8 (8.8–not reached)	9.4 (8.3–13.5)	11.6 (8.0–16.2)
TR (90% CI)		0.71 (0.44–1.11) *P* = 0.18	0.87 (0.71–1.09) *P* = 0.34
Response to treatment			
Complete response	2	0	0
Partial response	3	15	8
Stable disease	13	11	16
Progressive disease	13	6	9
Not evaluated	7	4	4
Duration of response^b^ (months)			
Median duration (90% CI)	3.8 (0.6–not reached)	3.6 (1.9–6.6)	4.6 (3.1–5.5)
ORR^c^, *n* (%)	5 *(13)*	15 (42) *P* = 0.01	8 (22) *P* = 0.34
Odds ratio (90% CI)	1.0	4.7 (1.7–13.2) *P* = 0.01	1.82 (0.6–5.2) *P* = 0.34

All *P*-values reported are two-sided; *P*-value <0.1 was considered to be significant.

a As the Cox model did not fit, the accelerated failure time model was used, as required in the analysis plan, giving time ratios. A TR above 1 implies that the covariate prolongs the time to event.

b Median duration of response from Kaplan–Meir graph of time from best overall response (whether complete or partial response) to relapse or death.

c ORR, the best overall response for each patient portrayed as the proportion of achieving complete response or partial response out of patients randomised.

CI, confidence interval; TR, time ratio; OR, odds ratio; *n*, number of patients in a group; ORR, objective response rate.

**Figure 2.**
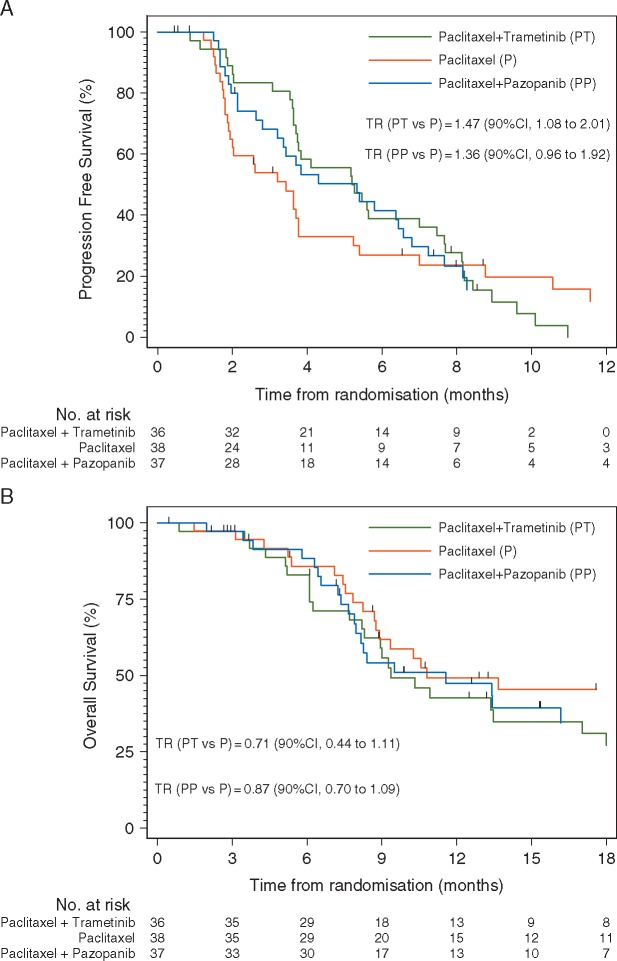
Kaplan–Meier curve for (A) progression-free survival and (B) overall survival with the time ratio (TR) calculated using a log-logistic accelerated failure time model.

A Cox regression was run for the PFS analysis and the proportional hazard assumption checked. As the assumption failed, Cox regression was not considered a valid method here and the results are not reported. Following the pre-specified statistical analysis plan, an AFT model was used instead to analyse PFS. PFS was significantly longer in the paclitaxel–trametinib arm than in single-agent paclitaxel (TR 1.47; 90% CI 1.08–2.01; two-sided *P *=* *0.04). There was no difference in PFS between the paclitaxel–pazopanib and single-agent paclitaxel arms (TR 1.36; 90% CI 0.96–1.93; two-sided *P *=* *0.14) (Figure 2A; Table 2).

ORR was significantly higher with paclitaxel and trametinib than with single-agent paclitaxel (42% versus 13%, two-sided *P *=* *0.01), but paclitaxel and pazopanib showed no improvement over single-agent paclitaxel (22% versus 13%, two-sided *P = *0.34) (Table 2).

The PFS rates at 6 months were 27%, 39%, and 41% in the single-agent paclitaxel, paclitaxel–trametinib, and paclitaxel–pazopanib groups, respectively. There was no significant difference in 6-month PFS rate between the paclitaxel–trametinib and single-agent paclitaxel arms (two-sided *P *=* *0.27) or the paclitaxel–pazopanib and single-agent paclitaxel arms (two-sided *P *=* *0.18).

After a median follow-up of 25.9 months (90% CI 20.1–32.9) and 66 deaths, the median OS was 10.8 months (90% CI 8.8–not reached) in the paclitaxel group, 9.4 months (90% CI 8.3–13.5) in the paclitaxel–trametinib group and 11.6 months (90% CI 8.0–16.2) in the paclitaxel–pazopanib group (Table 2). Due to the AFT model being required for PFS, it was also used for the OS analysis. The OS was not significantly different between the paclitaxel–trametinib and single-agent paclitaxel arms (TR 0.71; 90% CI 0.44–1.11; two-sided *P *=* *0.18*)* or paclitaxel–pazopanib and single-agent paclitaxel arms (TR 0.87; 90% CI 0.70–1.09; two-sided *P *=* *0.34) (Figure 2B; Table 2).

A sensitivity analysis repeating all tests using the per-protocol population gave broadly similar results to the intention-to-treat population, so is not reported in detail. As samples sizes were too small for the planned subgroup analysis comparing outcomes in *NRAS* mutant and wild-type, the results are not reported here.

### Safety

All of the 111 randomised participants who received treatment were included in the safety analysis. Almost all (109, 98%) experienced an AE. Rash was more prevalent in participants taking trametinib. Transaminitis and altered taste were more prevalent in those on pazopanib. Participants who were assigned targeted therapy experienced anorexia more frequently. Grade 3 or higher AEs were recorded in 9 (24%) of the 38 patients in the single-agent paclitaxel group, 27 (75%) of the 36 patients in the paclitaxel–trametinib group, and 29 (78%) of the 37 patients in the paclitaxel–pazopanib group (Table 3). Nineteen (54%) participants receiving paclitaxel–pazopanib stopped taking pazopanib due to toxicities, whereas 6 (18%) participants receiving paclitaxel–trametinib stopped taking trametinib.

**Table 3.** Incidence of treatment-related adverse events

AEs	Paclitaxel (*n *=* *38)	Paclitaxel+trametinib (*n *=* *36)	Paclitaxel+pazopanib (*n *=* *37)
	*n* (%)		
Any grade AEs	36 (95)	36 (100)	37 (100)
Grade ≥3 AEs	9 (24)	27 (75)	29 (78)
Fatal AEs	0 (0)	1 (4)	2 (5)
AE per patient for AEs affecting 15 or more patients^a^^,^^b^	*n* (*n* of grade 3 or more)		
Rash	6 (0)	35 (14)	9 (0)
ALT increased	1 (0)	2 (0)	12 (9)
Fatigue	19 (1)	24 (4)	18 (3)
Dyspnoea	8 (1)	10 (1)	8 (2)
Abdominal pain	7 (0)	11 (1)	14 (2)
Diarrhoea	17 (0)	24 (2)	20 (1)
Nausea	11 (0)	12 (0)	16 (1)
Anaemia	3 (0)	6 (2)	8 (0)
Alopecia	17 (1)	13 (0)	13 (0)
Anorexia	6 (0)	13 (0)	11 (0)
Constipation	11 (0)	13 (0)	2 (0)
Dysgeusia	3 (0)	4 (0)	12 (0)
Dyspepsia	7 (0)	4 (0)	4 (0)
Epistaxis	4 (0)	9 (0)	6 (0)
Headache	2 (0)	6 (1)	7 (0)
Vomiting	6 (0)	5 (0)	9 (0)
Cough	8 (0)	5 (0)	5 (0)

a The AE terms are ranked according to the highest frequency of grade ≥3.

b In any one row, a patient appears only once whether they had one episode of the AE or many.

AE, adverse events measured by Common Terminology Criteria for Adverse Events V4.03; ALT, alanine aminotransferase; n, number of patients in a group.

Seventy-two serious AEs (SAEs) were reported in 47 patients. The incidence of SAEs was higher when paclitaxel was combined with trametinib (47%) or pazopanib (67%) than when given alone (13%). Deaths during study treatment or within 35 days of the last study medication were reported for two participants on paclitaxel and pazopanib and one participant taking paclitaxel and trametinib. One participant in the paclitaxel–pazopanib arm died of pneumonia that was likely to have been related to paclitaxel-induced myelosuppression. Another participant in the same group died of bowel perforation related to disease progression, however, we were unable to exclude a contribution from pazopanib. One patient in the paclitaxel–trametinib arm died due to disease progression.

## Discussion

Although patients with *BRAF*-mutant melanoma have access to effective targeted treatments, these do not yet exist for *BRAF*-wt melanoma. Effective treatment options are urgently needed for patients with *BRAF*-wt melanoma, particularly those with rapidly progressive disease.

As melanoma is almost always associated with an activated MAPK pathway, even in tumours not driven by *BRAF* mutation, MEK inhibition is a promising therapeutic strategy. However, the activity of MEK inhibitors in *BRAF-*mutant melanoma has not been replicated in other genotypes [12]. Binimetinib showed an ORR of 20% and a median PFS of 4 months in *BRAF*-wt *NRAS*-mutant melanoma patients in a phase II trial [13]. However, in the randomised, phase III NEMO trial, the median PFS was only 2.8 months, ORR was 15%, and OS was not statistically significantly different from that achieved with dacarbazine chemotherapy [14].

Our study showed superior PFS for paclitaxel and trametinib compared with paclitaxel chemotherapy alone. These results were obtained even though half of the patients had elevated LDH and nearly 40% a mutation in tumour *NRAS*, both of which are associated with poor prognosis. The outcomes with paclitaxel alone were consistent with published data from larger studies, providing evidence that the improvements seen in PFS and ORR when trametinib was added cannot be explained by poor performance in the control arm [15, 16].

However, the significant difference in PFS did not translate into an improvement in OS. This is consistent with the modest durability of responses in all three study arms, at around 4 months. Post-progression treatment with immunotherapy may also have influenced this outcome. Most of the participants had not received prior systemic therapy, as there was limited availability of checkpoint inhibitors early in the life of this trial. The NEMO trial and a study of docetaxel with or without selumetinib in *NRAS*-mutant non-small-cell lung cancer also found a PFS advantage that did not yield an OS benefit [17]. Thus, the biological effects of MEK inhibition may be rapidly overcome by *BRAF*-wt tumours, perhaps due to the loss of negative feedback mechanisms under MEK inhibition.

The median PFS observed at 5.2 months when combining paclitaxel and trametinib was superior to that observed with binimetinib alone in phase II and III (NEMO) melanoma trials [13, 14]. The patient populations in the current study and NEMO trial were different, as we included patients with neither *NRAS-* nor *BRAF*-mutant melanoma. However, PACMEL included a higher proportion of participants with elevated LDH. The MEK inhibitor-chemotherapy combination regimen’s tolerability was similar to that of binimetinib alone, with 6 of the 33 (18%) participants discontinuing treatment because of toxicity in our paclitaxel–trametinib arm, compared with 20% of binimetinib-treated patients in the NEMO study.

The improved outcomes in the paclitaxel–trametinib arm came at the cost of more AEs than when using paclitaxel alone, with 75% of patients experienced a grade 3 or higher toxicity. The paclitaxel pazopanib combination also caused grade 3 or 4 AEs in three quarters of patients and over half of the participants had to discontinue treatment, mainly due to hepatotoxicity.

In conclusion, the PACMEL phase II trial found that adding the MEK inhibitor trametinib to paclitaxel chemotherapy significantly improved PFS and ORR in patients with *BRAF*-wt melanoma, but did not impact OS.

## Supplementary Material

Supplementary Data
