# Supplementary figures and images for: Paclitaxel with or without trametinib or pazopanib in advanced wild-type BRAF melanoma (PACMEL): a multicentre, open-label, randomised, controlled phase II trial
Source: Ann Oncol. 2018 Nov 14;30(2):317–24. doi: 10.1093/annonc/mdy500 (PMC6386028; doi:10.1093/annonc/mdy500)

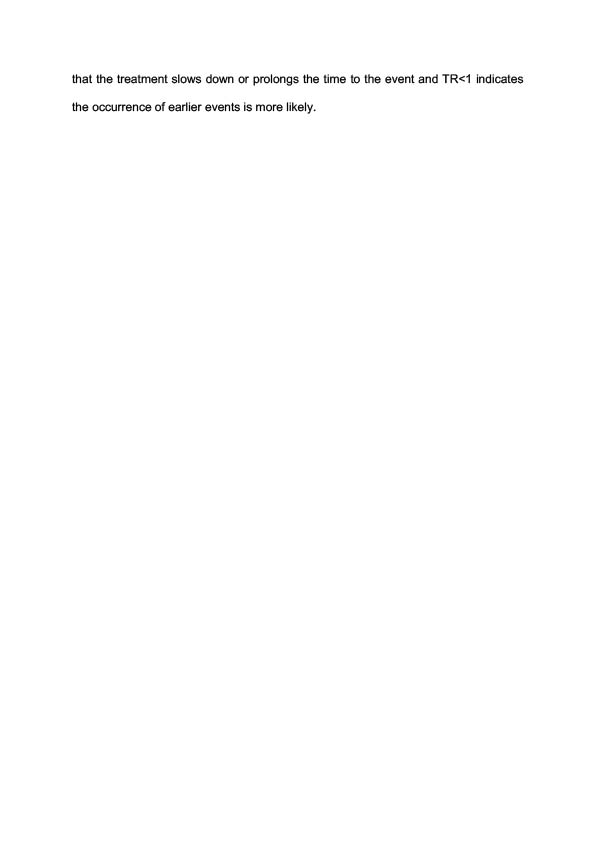

Supplement: Supplementary Data [file mdy500_supp.zip › mdy500-suppl_data/mdy500_Supplementary_Figure_S1_3.jpg]

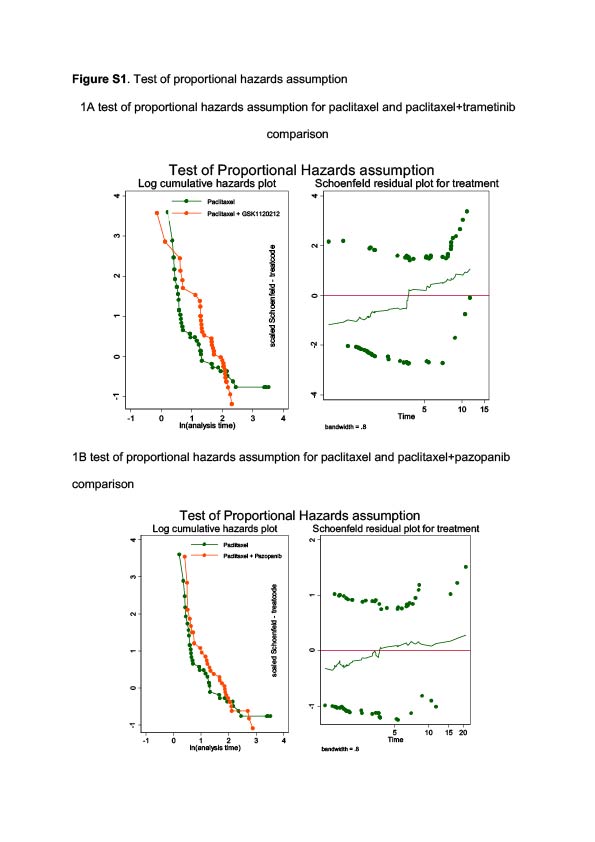

Supplement: Supplementary Data [file mdy500_supp.zip › mdy500-suppl_data/mdy500_Supplementary_Figure_S1_1.jpg]

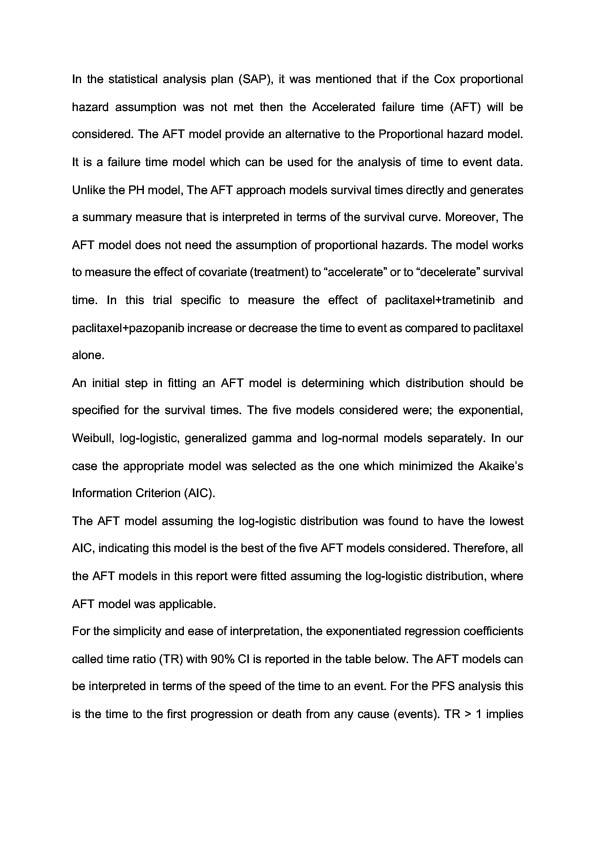

Supplement: Supplementary Data [file mdy500_supp.zip › mdy500-suppl_data/mdy500_Supplementary_Figure_S1_2.jpg]
